# Follow-up Results of Endovascular Aneurysm Repair Following Abdominal Visceral Debranching

**DOI:** 10.21470/1678-9741-2020-0705

**Published:** 2022

**Authors:** Didem Melis Oztas, Murat Ugurlucan, Omer Ali Sayin, Feza Ekiz, Yilmaz Onal, Metin Onur Beyaz, Muzaffer Umutlu, Mert Meric, Bulent Acunas, Ufuk Alpagut

**Affiliations:** 1 Cardiovascular Surgery Clinic, Istanbul Training and Research Hospital, Istanbul, Turkey.; 2 Department of Cardiovascular Surgery, Medical Faculty, Istanbul Medipol University, Istanbul, Turkey.; 3 Department of Cardiovascular Surgery, Istanbul Faculty of Medicine, Istanbul University, Istanbul, Turkey.; 4 Department of General Surgery, Istanbul Faculty of Medicine, Istanbul University, Istanbul, Turkey.; 5 Radiology Clinic, Fatih Sultan Mehmet State Hospital, Istanbul, Turkey.; 6 Department of Cardiovascular Surgery, Medical Faculty, Hatay Mustafa Kemal University, Hatay, Turkey.; 7 Department of Radiology, Istanbul Faculty of Medicine, Istanbul University, Istanbul, Turkey.

**Keywords:** Dissecting Aneurysm, Thoracic Aortic Aneurysm, Coronary Artery Bypass, Saphenous Vein, Polytetrafluoroethylene, Renal Artery

## Abstract

**Introduction:**

The aim of this study is to present a series of six cases with thoracoabdominal aneurysm treated with hybrid technique in our center.

**Methods:**

Between May 2015 and December 2018, the data of six patients with thoracoabdominal aneurysms and various comorbidities who underwent visceral debranching followed by endovascular aortic aneurysm repair were reviewed retrospectively.

**Results:**

Patients’ mean age was 65.3±19.6 years. All of them were male. Comorbidities were old age, congestive heart failure, coronary artery disease, chronic obstructive pulmonary disease, previous surgical interventions, and/or esophageal hemangioma. Except for one patient who underwent coronary artery bypass grafting (inflow was taken from ascending aorta), debranching was performed from the right iliac artery. Debranching of four visceral arteries (superior mesenteric artery, celiac trunk, and bilateral renal right arteries) was performed in three patients, of three visceral arteries (superior mesenteric artery, celiac trunk, right renal artery) was performed in one, and of two visceral arteries (superior mesenteric artery, celiac trunk) was performed in two patients. Great saphenous vein and 6-mm polytetrafluoroethylene grafts were used in one and five patients, respectively, for debranching. Endovascular aneurysm repair was performed following debranching procedures as soon as the patients were stabilized. In total, three patients died at the early, mid, and long-term follow-up due to multiorgan failure, pneumonia, and unknown reasons.

**Conclusion:**

Hybrid repair of thoracoabdominal aneurysms may be an alternative to fenestrated or branched endovascular stent grafts in patients with increased risk factors for open surgical thoracoabdominal aneurysm repair; however, the procedure requires experience and care.

**Table:** Abbreviations, Acronyms & Symbols

CABG	= Coronary artery bypass grafting	LRA	= Left renal artery
CAD	= Coronary artery disease	PTFE	= Polytetrafluoroethylene
Ch-EVAR	= Chimney EVAR	RA	= Renal artery
COPD	= Chronic obstructive pulmonary disease	RRA	= Right renal artery
CT	= Celiac trunk	SMA	= Superior mesenteric artery
EVAR	= Endovascular aneurysm repair	TEVAR	= Thoracic endovascular aneurysm repair

## INTRODUCTION

Conventional open surgery of the aortic arch and thoracoabdominal aneurysms is the gold standard therapy, however, it still carries serious mortality and morbidity risks^[1]^. Consequences of this surgery include mortality as well as visceral organ dysfunction, paraplegia, and complications of cardiopulmonary bypass^[2]^. Complication rates range between 5-19%, including spinal cord ischemia (2.7-13.2%) and renal failure (4.6-5.6%)^[1]^.

There is ongoing search for alternatives to open surgical techniques for thoracoabdominal aneurysm repair using advanced technology, but none has been proven to be as effective. Hybrid techniques preceded by debranching procedures are considered as one of the solutions in especially elderly patients or patients with comorbidities — complex and extensive pathologies^[1]^. The technique has been also used in the treatment of Types A and B dissections to enable new convenient landing zones for endovascular repair with debranching of branches of aortic arch and visceral arteries^[3]^. It is especially beneficial in emergency cases who have challenging anatomies and are not appropriate for sole endovascular procedures due to anatomical limitations^[1]^.

Spinal cord ischemia and paraplegia are two of the most unwanted complications of open repair of thoracoabdominal aortic aneurysms^[1]^. The hybrid repair offers decreased rates of spinal cord ischemia and paraplegia. The renovisceral debranching in hybrid repair prevents perioperative hypotension and long ischemia time of the visceral arteries^[1]^. Hybrid repair is also convenient in recurrent thoracoabdominal aneurysms. Reoperations are challenging due to scarred tissue and extensive adhesions^[4]^.

In this manuscript, we present the follow-up results of our patients with thoracoabdominal aneurysms who underwent abdominal visceral debranching followed by endovascular aneurysm repair (EVAR). These patients had various comorbidities or history of previous endovascular or surgical procedures. We aimed to present the feasibility of hybrid repair in different patients with various comorbidities.

## METHODS

Between May 2015 and December 2018, six patients with the diagnosis of thoracoabdominal aneurysms underwent debranching procedures of visceral arteries prior to endovascular aortic aneurysm repair. These patients had increased risks for conventional surgical thoracoabdominal replacement due to old age, comorbidities, or history of previous aneurysm repair with open or endovascular techniques. Other patients, who underwent conventional open surgical repair or full endovascular interventions (with non-branched straight grafts), were excluded from the study. The study was conducted at the Istanbul Faculty of Medicine, Istanbul University. All the authors either used to work or are still working at the aforementioned institution.

### Surgical Technique and Endovascular Aneurysm Repair

**Fig. 1:**
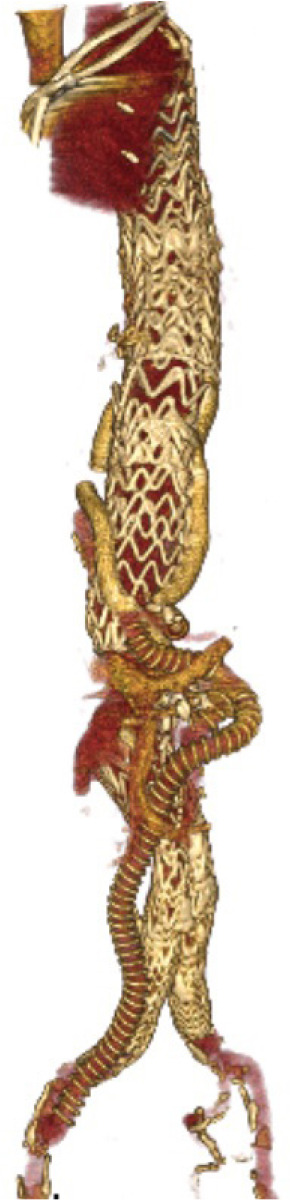
Postoperative computed tomography angiography of the patient.

All debranching procedures were performed with general anesthesia. An extended median laparotomy was performed and, depending on the need, the abdominal aorta, superior mesenteric artery (SMA), celiac trunk (CT), right and left renal arteries (RRA/LRA), or bilateral iliac arteries were dissected. Appropriate length and number of debranching grafts were prepared by anastomosing 6-mm separate grafts to an 8-mm body graft. The tip of the 8-mm graft is preferred to debranch the SMA, whereas the side branches are used for CT and bilateral renal artery (RA) when needed. Following systemic heparinization, the proximal end of the graft was anastomosed to the right iliac artery (Figure 1).

**Fig. 2:**
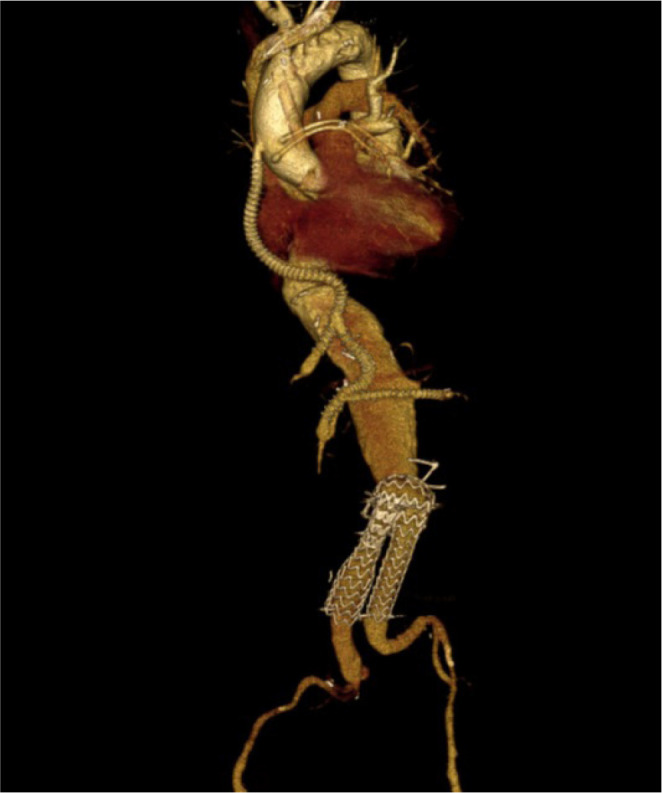
Postoperative computed tomography angiography of the patient.

The patient who had coronary artery disease underwent triple vessel coronary artery bypass grafting followed by abdominal debranching (Figure 2). The proximal part of the 8-mm polytetrafluoroethylene (PTFE) graft was anastomosed to the ascending aorta. The graft was carefully passed through the diaphragm, and the distal side of the graft was anastomosed to SMA. The other 6-mm PTFE graft branches were anastomosed to RRA and CT.

In one patient, the great saphenous vein was preferred for the debranching of RAs as they were very fragile.

Debranching of four visceral arteries (SMA, CT, RRA/LRA) was performed in three patients, of three visceral arteries (SMA, CT, RRA) was performed in one patient, and of two visceral arteries (SMA, CT) was performed in two patients.

For the thoracic endovascular aneurysm repair (TEVAR), the right femoral artery was prepared surgically with spinal anesthesia. Super Stiff 0.035-inch guidewire (Back-up Meier, Schneider Co.; Blach, Switzerland) was positioned at the ascending aorta through the longitudinal arteriotomy. A 5F sheath was inserted percutaneously to the left femoral artery to enable directing a 5F pigtail catheter for angiographic monitoring of the stent graft position. Following systemic 5,000 units of heparin, the endovascular stent graft delivery system (Endurant Medtronic Endovascular, Santa Roja, California, United States of America) was inserted through a longitudinal incision in the right common femoral artery and positioned at the thoracic aorta. The stent graft was then expanded. Additional stent grafts were implanted to exclude the aneurysms completely with safe landing zones when needed. The femoral artery was reconstructed primarily or with a patch when needed.

## RESULTS

The mean age of the patients was 65.3±19.6 years (range: 27-84 years, median: 70.5). All of them were male and had thoracoabdominal aneurysms. Comorbidity factors were old age, congestive heart failure, coronary artery disease, chronic obstructive pulmonary disease, previous surgical interventions, and/or esophageal hemangioma. In addition, three patients had hypertension, two patients had diabetes mellitus, two patients had chronic obstructive pulmonary disease, one patient had Marfan syndrome, and one patient had gastric problems and esophageal hemangioma.

**Fig. 3:**
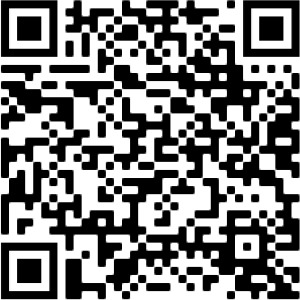
Preoperative computed tomography angiography of giant thoracoabdominal dissecting aneurysm in the patient with Marfan syndrome.

**Fig. 4:**
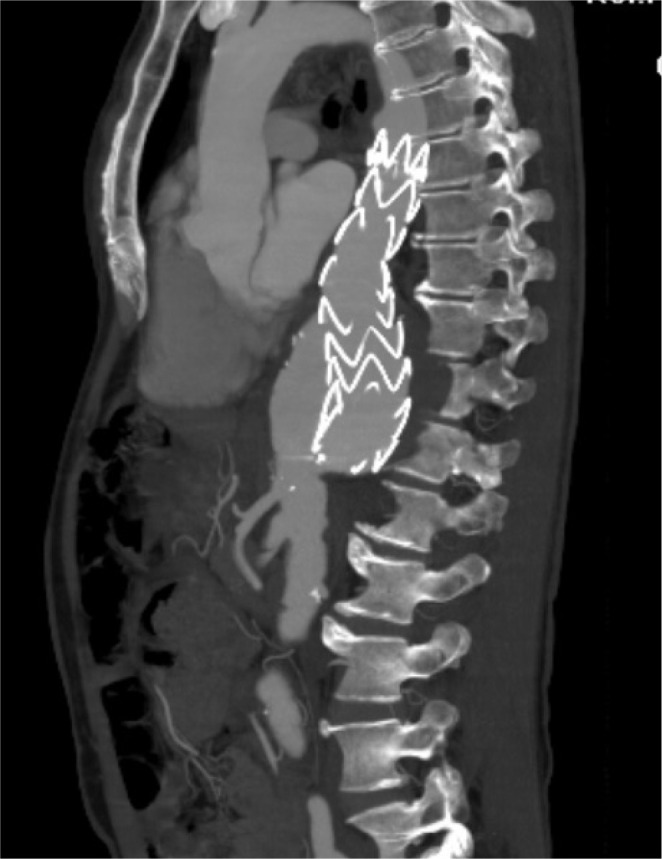
Preoperative computed tomography angiography of increasing diameter of aneurysm and Type 1b endoleak in the distal part of the stent graft in the patient with history of thoracic endovascular aneurysm repair.

**Fig. 5:**
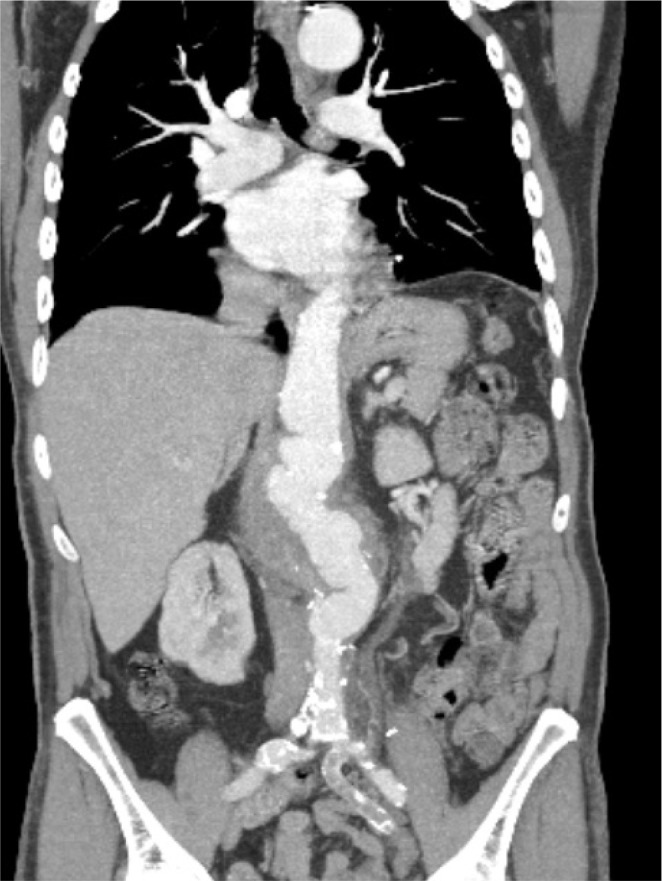
Preoperative computed tomography angiography of thoracoabdominal aneurysm in the patient with severe coronary artery disease.

The patient with Marfan syndrome was a 27-year-old male and had undergone Bentall-De Bono procedure and aortic arch and infrarenal abdominal aortic replacements. He presented with giant thoracoabdominal dissecting aneurysm (Figure 3). Although conventional surgical thoracoabdominal therapy was the ideal option for this particular case, he strongly refused the open surgical repair^[2]^. One patient had history of Chimney EVAR (Ch-EVAR) (including SMA and CT) and was admitted with Type 1b endoleak. An 84-year-old patient had a history of multi-layer flow modulating stent (MFMS, Cardiartis, Belgium) insertion to treat thoracoabdominal aneurysm, and enlargement of the aneurysm sac was detected in the follow-up (Video 1). Another patient with thoracoabdominal aneurysm had a history of TEVAR for the treatment of juxtaceliac descending aortic aneurysm and presented with increasing diameter of aneurysm secondary to Type 1b endoleak from the distal end of the stent graft (Figure 4, Video 2). The last patient had life-threatening coronary lesions as well as very large thoracoabdominal aneurysm causing severe back pain (Figure 5). The demographic features of the patients are presented on Table 1.

**Table 1:** Demographic features of the patients.

Demographic features	Patients (n=6)
Male/female	6/0
Mean age (years)	65.3±19.6
Hypertension	3
Diabetes mellitus	2
Renal failure	-
Coronary artery disease	4
Smoker/ex-smoker	3/1
Chronic obstructive pulmonary disease	2
Gastritis/esophageal hemangioma	1
Connective tissue disorder	1

**Video 1:**
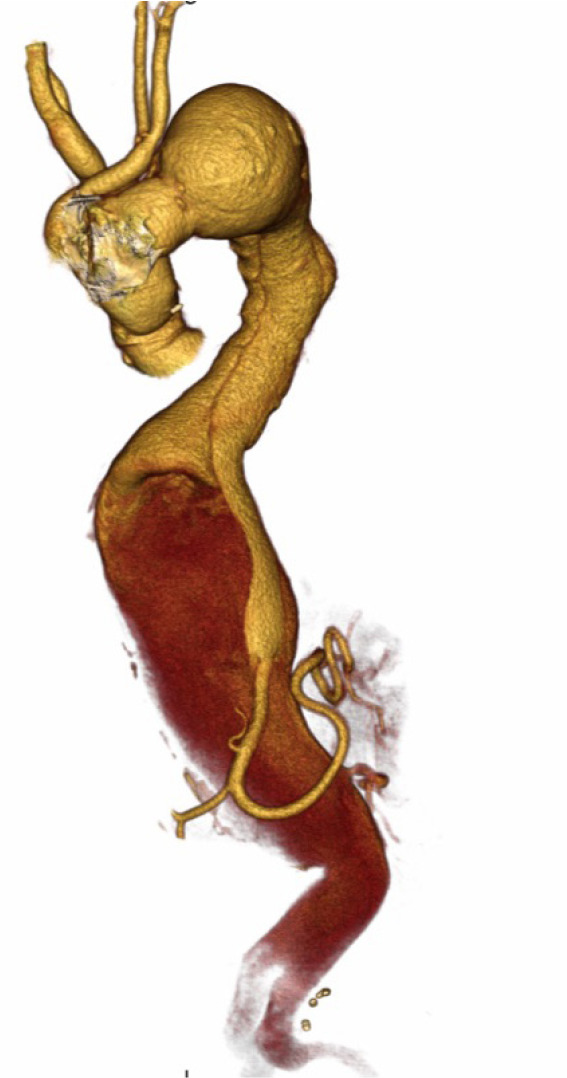
Digital subtraction angiography of the patient with history of flow modulator stent insertion before and after endovascular stent graft repair.

**Video 2:**
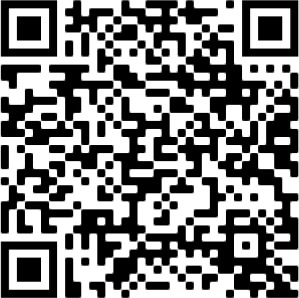
Digital subtraction angiography of the patient with history of thoracic endovascular aneurysm repair, presented with increasing diameter of aneurysm and Type 1b endoleak in the distal part of the stent graft.

All patients were evaluated by a team composed of anesthesiologists, cardiologists, radiologist, and cardiovascular surgeons. The American Society of Anesthesiologists (or ASA) classification scores of the patients ranged between 3 and 4. Due to the high risk, hybrid repair was preferred in all patients, and EVAR was performed following abdominal visceral debranching in a staged manner.

All patients received a combination of 100 mg of aspirin and 75 mg of clopidogrel for three months, followed by a single lifelong antiplatelet. At least 20 mg of atorvastation was prescribed for one year, and it was continued depending on the cholesterol levels in the long term. The follow-up period for the patients is adjusted as: early period, hospital stay and within one month of procedure; mid-term follow-up, the first year of procedure; and long-term follow-up, beyond the first year.

Except for one patient, debranching surgery and EVAR were performed as staged procedures in all of the patients. The mean period between abdominal debranching and endovascular intervention was 7.4±1.14 days (range: 5-9 days, median: seven). Mortality did not occur in any patient during procedures.

Bleeding requiring re-exploration occurred in one patient on the first postoperative day. All patients were taken to the intensive care unit following debranching and endovascular procedures. Except for one patient with severe chronic obstructive pulmonary disease, all patients were extubated in the first day of intensive care unit. The mean intensive care unit stay was 3±0.8 days (range: 2-4 days, median: three), and mean hospital stay was 11±0.8 days (range: 10-13 days, median: 11), except for the patients who died in the early and mid-term follow-up. Neurological morbidity was not observed during the procedures or follow-up period.

An 84-year-old patient with chronic obstructive pulmonary disease who underwent thoracoabdominal EVAR procedure following debranching surgery could not be weaned off the mechanical ventilator although extubation was attempted two times. Hemodialyses were required due to renal failure, and the patient died at the hospital on the 12^th^ day.

We postponed the TEVAR procedure in a patient who underwent coronary artery bypass grafting and debranching procedure simultaneously. His creatinine levels and liver functions deteriorated early after surgery but returned to normal within one week. TEVAR was then performed, however, acute renal failure requiring hemodialysis occurred. The intensive care unit stay was prolonged in this patient. As soon as the need for hemodialysis was over, the patient was taken to the ward in good conditions. Ward stay was complicated with pneumonia followed by renal failure which ended up with multiorgan failure and death after one month.

The patient with Marfan syndrome underwent endovascular thoracoabdominal stent graft implantation between the aortic arch and infrarenal abdominal aortic grafts. The patient with previous history of Ch-EVAR underwent stent graft implantation covering Chimney graft and thoracic grafts to eliminate endoleak. The patient with a history of TEVAR and Type 1b endoleak received extension of the stent graft to the orifice level of the RA (Video 2). In the patient with esophageal hemangioma, stent graft was extended from the thoracic aorta to the infrarenal abdominal aorta. All patients were followed up in the intensive care unit and ward without any event.

In the mid-term follow-up (between six and nine months), thoracoabdominal computerized tomography angiography was performed in all patients. In one patient, the graft of RRA had 30% stenosis but his creatinine levels did not increase, and in the late follow-up period, the stenosis did not increase, and renal functions were normal. Except for this case, all grafts were patent in all patients. In one patient who had previous history of TEVAR, Type 2 endoleak was detected; however, the aneurysm size did not increase in the follow-up.

In the long-term follow-up, we lost contact with the Marfan syndrome patient. The remaining patients were followed-up and remained symptom free and in good conditions with patent debranching grafts and excluded aneurysm. The aneurysm of the patient with Type 2 endoleak remained with 4.6 cm in diameter in the abdominal aorta, and further intervention is not planned in the current stage. The preoperative, operative, and postoperative features of the patients are summarized in Table 2.

**Table 2:** Preoperative, operative, and postoperative features of the patients.

Patients	Comorbidities and history	Presentation	Debranching procedure	Endovascular intervention	Early follow-up	Mid-term follow-upw	Long-term follow-up
1	- Marfan syndrome. - Bentall-De Bono procedure, aortic arch and infrarenal abdominal aortic replacements.	- Giant thoracoabdominal dissecting aneurysm. - Crawford Type 2 thoracoabdominal aneurysm.	Debranching of SMA, celiac trunk, right and left renal arteries with 6-mm PTFE grafts taking inflow from right common iliac artery.	TEVAR	Uneventful	Uneventful	The contact was lost.
2	- Hypertension, diabetes mellitus, smoking. - Ch-EVAR (including superior mesenteric artery and celiac trunk).	- Type 1b endoleak in distal portion of stent graft. - Crawford Type 5 thoracoabdominal aneurysm.	Debranching of SMA and celiac trunk with 6-mm PTFE grafts taking inflow from right common iliac artery.	Stent graft implantation covering Chimney graft and thoracic grafts to eliminate endoleak.	Uneventful	Uneventful	Uneventful
3	- Hypertension, COPD, CAD, smoking. - Flow modulator stent insertion to treat thoracoabdominal aneurysm.	- Enlargement of aneurysm sac. - Crawford Type 5 thoracoabdominal aneurysm.	Debranching of SMA, celiac trunk, and right renal artery with 6-mm PTFE grafts taking inflow from right common iliac artery.	TEVAR	He could not be weaned off. Hemodialyses were required due to renal failure, and the patient was lost in 1 month in intensive care unit.		
4	- Diabetes mellitus, CAD, ex-smoker, esophageal hemangioma. - TEVAR.	- Increasing diameter of aneurysm and Type 1b endoleak in the distal part of the stent graft. -Crawford Type 1 thoracoabdominal aneurysm.	Debranching of SMA and celiac trunk with saphenous grafts taking inflow from right common iliac artery.	TEVAR extension	Uneventful	Type 2 endoleak was detected.	The aneurysm size remained 4.6 cm in diameter in abdominal aorta.
5	Hypertension, CAD, smoking.	- Coronary artery disease. - Crawford Type 3 thoracoabdominal aneurysm.	CABG × 3, debranching of SMA, celiac trunk, right and left renal arteries with 6-mm PTFE grafts taking inflow from ascending aorta.	TEVAR	Hemodialysis required due to acute renal failure, then the requirement of hemodialysis disappeared.	Pneumonia, hemodialysis requirement. He was lost after one month because of multiorgan failure.	
6	CAD, COPD.	- Crawford Type 2 thoracoabdominal aneurysm.	Debranching of SMA, celiac trunk, right and left renal arteries with 6-mm PTFE grafts taking inflow from right common iliac artery.	TEVAR	Uneventful	The graft of right renal artery had 30% stenosis, but his creatinine levels did not increase.	The stenosis did not increase, and renal functions were normal.

CABG=coronary artery bypass grafting; CAD=coronary artery disease; Ch-EVAR=Chimney EVAR; COPD=chronic obstructive pulmonary disease; PTFE=polytetrafluoroethylene; SMA=superior mesenteric artery; TEVAR=thoracic endovascular aneurysm repair

## DISCUSSION

Conventional open surgical repair of thoracoabdominal aneurysms has high mortality and morbidity rates despite advances in surgical field. Morbidities include paraplegia, stroke, and visceral, intestinal, and renal ischemia, as well as dialysis requirement due to renal failure, mostly as a result of distal aortic ischemia^[5]^.

Although EVAR is still not the gold standard method for aneurysm repair, it is associated with decreased blood loss and shorter recovery time when compared with open surgical therapy^[6]^. It does not require aorta cross-clamping. Also, 30-day all-cause mortality and perioperative morbidity rates are found to be lower in endovascular procedures^[6]^. Despite the increasing number of percutaneous treatment options for thoracic and abdominal aortic aneurysms in the last few decades, the procedure is not always suitable due to anatomical limitations. Open surgical repair with established long-term follow-up results is still the gold standard for complex aneurysms without appropriate neck for landing zone for EVAR^[7]^.

Conventional EVAR or TEVAR is not suitable for patients with thoracoabdominal aneurysms extending to the visceral arteries^[7]^ or suprarenal abdominal aortic aneurysms^[8]^. The need for special branched or fenestrated grafts and/or hybrid procedures arise. When the aneurysm is at the vicinity of visceral arteries or if there is no safe proximal or distal landing zones, endovascular procedures may fail, and open surgery may be required^[9]^. In such circumstances, when open surgery carries high risks, hybrid procedures are also valid alternatives.

Custom-made fenestrated stent grafts may be preferred in aortic aneurysms and in branched zones of the aorta. The disadvantage of this technique is the waiting time for the manufacturing of the stent graft, which is not feasible in emergency cases. Cost is another issue. Intraoperative fenestration is another technique, however, these methods require experience and learning time to be performed as a practical and successful method^[10]^. Snorkel and Chimney techniques may also be applied, but they were found to be associated with higher Type 1 endoleaks and complication rates than fenestrated EVAR. In patients with acute conditions and challenging anatomies, debranching provides fast and safe treatment allowing overstenting of major arteries^[11]^. We have preferred hybrid repair in our patients due to availability of the branched or fenestrated stent graft options, long waiting duration for custom stent grafts, anatomical challenges, and cost issues related with social security services reimbursement.

The hybrid procedure was described first by Quinones-Baldrich et al. in 1999. The aim of this procedure was to avoid aortic cross-clamping, thoracotomy, single-lung ventilation requirement, and prolonged ischemia of lower half of the body^[5]^. Hybrid repair is mainly preferred in patients with comorbidities such as older age, congestive heart failure, decreased pulmonary capacity, and renal pathologies^[5]^, or for patients with history of previous surgery^[7]^. Although the debranching procedure requires a major laparotomy, it eliminates the need for cardiopulmonary bypass. Also, the procedure generally is not lengthy for every anastomosis individually, and ischemia period of each organ is quite short^[12]^. The inflow of debranching procedures may be provided from the iliac arteries, abdominal aorta, or a previously implanted intra-abdominal vascular graft^[13]^.

When compared with open surgical repair, hybrid repair was found to be associated with lower morbidity and mortality rates^[4]^. One of the largest studies in the literature was conducted by Drinkwater et al.^[14]^. The incidence of technical success was 93%, of permanent paraplegia was 8%, and of 30-day mortality was 15% in this multicenter study among 107 patients. The graft patency was 86% at 30 days, and initial endoleak rate was 33%. Although hybrid therapy was also associated with significant risk of mortality and morbidity according to their results, studies have shown that morbidity rates were 19% to 23% in open thoracoabdominal aneurysm repair^[14]^. Also, these patients had additional risks due to comorbidities. Again, in a meta-analysis of 19 studies among 507 patients, 30-day mortality was 12%, permanent paraplegia was 4.5%, and renal insufficiency was 8.8%. Mean follow-up period was 34 months, and the graft patency, endoleak, and reintervention rates were 96%, 23%, and 27%, respectively^[15]^. These results showed that hybrid repair had been superior to open surgical repair in especially high-risk patients^[15]^.

Open surgical repair was regarded as a high risk in our cohort. Hybrid repair was considered as a relatively less invasive method and proposed to be associated with lesser postoperative complications associated with mechanical ventilation and renal or other organ systems in our fragile patient population^[7]^. On the other hand, the disadvantages of hybrid repair include complications of both open and endovascular procedures^[7]^. Endoleaks, migration of endograft, kinking and/or stenosis in a limb of endograft and graft infections are complications of endovascular repair^[6]^. Requirement of reintervention rates may be approximately 19% to 24% of patients who receive endovascular abdominal and thoracic aortic aneurysm repairs, respectively^[6]^. Endoleak or enlargement of sac may be seen despite successful procedure and graft occlusion; infections are other complications of open debranching surgery^[7]^.

There is a sparse number of studies in the literature comparing total endovascular approaches and hybrid repair for the treatment of thoracoabdominal aneurysm. In a study conducted by Tsilimparis et al.^[16]^, in which fenestrated-branched endografts and visceral debranching plus stenting (hybrid) for complex aortic aneurysm repair were compared, hybrid repair was found associated with higher early mortality rate. Acute renal failure leading to renal failure and dialysis requirement at discharge (2.6% *vs.* 18%), pulmonary complications leading tracheotomy (0% *vs.* 9%), and mesenteric ischemia (3% *vs.* 23%) were more common in the hybrid group than in the endovascular group. The paraplegia rate was higher in the endovascular group. The increased early graft failure in the hybrid group was found associated with high rate of bypass occlusions. The major cause of mesenteric ischemia was occlusion. Patients with connective tissue disorders and urgent cases who were not suitable for endovascular procedures had undergone hybrid repair. Although the major cause leading to early graft failure could not be identified, it was thought that atherosclerosis (in old patients) and dissection of vessels, which is common in patients with connective tissue disorder, might have been related with high rates of occlusions in the authors’ series^[16]^.

The correct positioning and debranching of grafts are important for graft patency. Following bowel reposition, kinking may be seen in anastomosis sites or grafts may be angulated or twisted. Stenosis or dissection of visceral vessels are other complications of debranching. A self-expanding stent may be used in the treatment of complications related to debranching grafts^[17]^. Also, the graft which is used for the debranching of hepatic artery is generally placed in antepancreatic route in order to avoid the risk of pancreatic injury during a posterior tunnel^[17]^. In our series, all the grafts were anatomically positioned over the aorta, retropancreatically, and at the posterior retroperitoneum.

In some series, the incidence of visceral graft patency was found to be 97% at 19.3 months, and in the literature, 30-day mortality was detected between 0 to 34%^[11]^. In the study conducted by Shaherdan et al., five-year primary patency rate was 86% in 46 patients with 164 grafts. Individually, patency rates of RRA, hepatic artery, LRA, and SMA had been found to be 69%, 100%, 87%, and 88%, respectively^[17]^. Again, in the meta-analysis performed by Canaud et al., the rates of primary patency were quite high, with 94.7% in a mean follow-up period of 26.2 months[17].

The benefit of staged procedures is controversial, and there is no evidence about safety of a two-stage procedure in the literature when planning hybrid aneurysm repair. Some authors recommend one-stage strategy due to the risk of aneurysm rupture during the waiting period and the possibility of refusing the patient endovascular intervention. Also, some authors may prefer transabdominal approach to insert endovascular stent graft to avoid damaging of the bypasses during the intervention with transfemoral access. However, two-stage procedure is mostly preferred due to certain advantages. The staged procedure is used in elective patients to reduce the operation time, requirement of blood and blood transfusions, and complications associated with respiratory and neurologic systems^[14]^. Also, it protects kidneys from contrast nephropathy immediately following renal ischemia occurring during abdominal debranching procedure. The staged procedure enables haemodynamic stabilization and decreased risk of spinal ischemia due to avoiding hypotension and gives an opportunity to evaluate spinal cord blood flow^[1]^.

Our patients had certain comorbidities, and five of six patients had a history of previous surgery or endovascular interventions. The open surgical repair had high risk of mortality and morbidity, also, the patient with Marfan syndrome had strongly refused the standard open surgical repair. Total endovascular repair could not be performed in our patients due to anatomical challenges as well as various other reasons like cost and ready availability issues. Hence, after careful examination, we have decided for the hybrid repair in these patients. We performed a staged procedure in all patients, except for one, with rapidly increasing size of aneurysm.

We have preferred saphenous vein graft in one patient because of small size and fragility of the arteries. In general, we preferred ringed PTFE grafts to prevent compression of intra-abdominal structures to the grafts. Due to the angle of the graft, debranching graft of the RRA is more likely to be stenosed when compared with other branches^[17]^. We have detected an insignificant stenosis in the debranching graft of RRA in one patient, however, the creatinine levels did not increase, or the renal functions did not impair, in the follow-up period.

In our cohort, one patient died in the early follow-up period, one patient died due to multiorgan failure in mid-term follow-up, and one patient died in long-term follow-up due to unknown reasons. We have not observed any neurological morbidities, which is not underestimated in conventional open surgical repairs.

### Limitations

The major limitations of our study were small size of the cohort and relatively short follow-up period (36 months). Additionally, the retrospective nature of the study plan may be regarded as another limitation.

## CONCLUSION

Hybrid repair of thoracoabdominal aneurysms may be safer for the treatment of patients with comorbidities and previous history of surgical or endovascular treatment of the aorta. In the patients with high surgical risk and who are not suitable for total endovascular approaches, depending on the patient- or center-related factors, the hybrid approach offers an alternative to the conventional open surgery. The results of hybrid repair depend on both patient and physician-related factors. Although the hybrid repair is less invasive than conventional open surgery, it should be noted that the procedure still carries certain risks. Long-term data from multicenter studies are warranted in order to establish treatment strategies for patients with challenging comorbidities who have thoracoabdominal aneurysms requiring treatment.
